# 

**Published:** 1870-07

**Authors:** 


### ARTICLE V.

### *The Fourth Annual Meeting of the Tennessee Dental Association.*

The fourth annual meeting of the Tennessee Dental Association, will be held at Murfreesboro, Tenn., on the July 28th, 1870. The following are the officers:

*President*, S. J. Cobb, Nashville, Tenn.; *First Vice President*, J. C. Ross, Nashville, Tenn.; *Second Vice President*, M. McCarty, Pulaski, Tenn.; *Recording Secretary and Treasurer*, H. M. Acree, Clarksville, Tenn.; *Corresponding Secretary*, C. P. Baird, Nashville, Tenn.

The following Committees were appointed at the last meeting:

*Committee on Diseased Antrum.*—W. H. Morgan, Nashville, Tenn.; W. R. Johnston, Columbia, Tenn.

*Committee on Diseased Gums.*—S. H. Beard, Murfreesboro, Tenn.; R. R. Freeman, Nashville, Tenn.

*Committee on Indication for, and Extracting Teeth.*— L. C. Chisholm, Nashville, Tenn. ; L. Augspath, Helena Ark.

*Committee on Preservation of Children's Teeth.*—E. C. Ford, Sommerville, Tenn.; L. T. Gunn, Nashville, Tenn.

*Committee on Dental Physiology.*—W. T. Arrington, Memphis, Tenn.; A. F. Claywell, Lebanon, Tenn.

*Committee on Voluntary Essays.*—C. P. Baird and L. C. Chisholm, Nashville, Tenn.

*Committee on Filling Teeth.*—J. A. Arrington, Jackson, Tenn.; Alex. Hartman, Murfreesboro, Tenn.; J. T. Cazier, Jonesboro, Tenn.; C. A. Jordan, Huntsville, Ala.

*Committee on Teaching Students.*—C. P. Baird and J. C. Ross, Nashville, Tenn.

*Committee on Dental Pathology.*—M. McCarty, Pulaski, Tenn.; J. B. Wasson, Memphis, Tenn.

*Committee on Dental Neuralgia.*—S. P. Cutler, Holly Springs, Miss.; J. Fouche, Knoxville, Tenn.

*Committee on Contour Fillings, Sponge Gold, and Heavy Foil.*—E. W. Sawyer, Memphis, Tenn. ; J. T. Cazier, Jonesboro, Tenn.

*Committee on Mechanical Dentistry.*—E. S. Chisholm, Tuscumbia, Ala.; L. C. Chisholm and R. Russell, Nashville, Tenn.; T. E. Beeeh, Franklin, Tenn.

---

### SELECTED ARTICLES.

---

